# Hepatitis B, Hepatitis C and HIV-1 Coinfection in Two Informal Urban Settlements in Nairobi, Kenya

**DOI:** 10.1371/journal.pone.0129247

**Published:** 2015-06-12

**Authors:** Glennah Kerubo, Samoel Khamadi, Vincent Okoth, Nyovani Madise, Alex Ezeh, Ziraba Abdalla, Matilu Mwau

**Affiliations:** 1 Institute of Tropical Medicine and infectious Diseases, Jomo Kenyatta University of Agriculture and technology, Nairobi, Kenya; 2 Centre for Virus Research, Kenya Medical Research Institute, Nairobi, Kenya; 3 Centre for Infectious and Parasitic Disease Control Research, Kenya Medical Research Institute, Busia, Kenya; 4 African Population Health Research Centre, Nairobi, Kenya; University of Rome Tor Vergata, ITALY

## Abstract

**Background:**

HIV-1 and Hepatitis B and C viruses coinfection is common in Sub-Saharan Africa due to similar routes of transmission and high levels of poverty. Most studies on HIV-1 and Hepatitis B and C viruses have occurred in hospital settings and blood transfusion units. Data on Hepatitis B and C viruses and HIV-1 coinfection in informal urban settlements in Kenya are scanty, yet they could partly explain the disproportionately high morbidity and mortality associated with HIV-1 infections in these slums.

**Objectives:**

The objective of this study was to determine the prevalence of HIV and Hepatitis B and C dual infection in urban slums in Nairobi.

**Methods:**

Blood samples were collected from residents of Viwandani and Korogocho between 2006 and 2007. A structured questionnaire was used to obtain socio-demographic data from participants. Samples were screened for Hepatitis B surface antigen (HBsAg), anti-HCV and anti-HIV-1. Statistical analysis was done using STATA.

**Results:**

Samples were successfully collected from 418 (32%) men and 890 (68%) females. The HIV-1, HBV and HCV prevalence was 20.4%, 13.3% and 0.76% respectively at the time of the study. Of the 268 (20.4%) HIV-1 positive participants, 56 (4.26%) had HBV while 6 (0.46%) had HCV. Of the 1041 HIV-1 negative participants, 117 (8.9%) had HBV while 4 (0.31%) had HCV. Only two people (0.15%) were co-infected with all the three viruses together.

**Discussion:**

The odds of getting hepatitis infection were higher in HIV-1 participants (for HBV OR 2.08,p<0.005 and for HCV OR 5.93, p<0.005). HIV prevalence rates were similar in both informal settlements. HIV infection was highest in age group 35-39 years and among the divorced/separated or widowed. Prevalence of all viruses was highest in those who did not have any formal education.

**Conclusion:**

The HIV prevalence in these informal settlements suggests a higher rate than what is observed nationally. The prevalence rates of HBV are significantly higher in the HIV-1 positive and negative populations. HCV as well as triple HIV-1, HBV and HCV coinfection are uncommon in Korogocho and Viwandani. This clearly indicates the need for HIV-1 control programmes and hepatitis B virus vaccination to be promoted through public awareness as preventive strategy.

## Introduction

Hepatitis B and C virus infections are the leading causes of liver disease and liver related deaths among those living with HIV infection, potentially due to the shared routes of transmission [1,2,3]. Worldwide, HBV accounts for about 370 million chronic infections, HCV for an estimated 130 million, and HIV for about 40 million. About 2–4 million people infected with HIV have chronic HBV co-infection and 4–5 million have HCV co-infection [4, 5]. However, these prevalence rates vary greatly from one region to another and over time. In Africa, HBV, HCV and HIV infections are considered to be endemic, but their rates are highly variable among the African countries. HBV and HCV prevalence rates range from 3–20% and 1–26%, respectively [4, 6,7]. Furthermore, over 63% of those infected with HIV worldwide reside in Sub-Saharan Africa. In Kenya, there is paucity of information on the prevalence of HBV, HCV and HIV-1 co-infections in informal urban settlements. Studies carried out on outpatients in three district hospitals showed that 11.4% were positive for HBsAg [8]. Another study done on blood donors in Nairobi indicate anti-HCV rate to be 1.8% [9]. However, one limitation of the studies done in Kenya is that they conducted in selected group of people with higher risk factors such as blood donors, drug addicts, commercial sex workers (CSWs) or hospitalised patients [10].

In developing countries, liver disease due to chronic HBV and/or HCV has become a growing problem, particularly in those infected with HIV. Therefore, it is important to document HIV co-infections in regions with high hepatitis chronicity and HIV infection rates [11,12]. Indeed, HIV accelerates the progression of chronic liver diseases related to HBV and HCV. Furthermore, most HIV patients are usually co-infected with viral hepatitis, which means that liver diseases will likely emerge as significant causes of morbidity and mortality among HIV infected individuals in Africa, similar to the trend worldwide. In Kenya, data from a previous study suggests that the HIV prevalence rate in informal settlements is higher than the national average [13]. There is anecdotal evidence to suggest that HIV-1 infected persons in the slums have a higher morbidity and mortality, often due to liver failure. The objectives of this study were to determine the prevalence rates of HBV, HCV and HIV in two informal settlements and to analyse the risk factors associated with co-infection with these viruses.

## Materials and Methods

### Institutional context

This study was conducted as part of a collaborative agreement between Kenya Medical Research Institute [KEMRI] and African Population Health research Centre [APHRC]. It was part in the Nairobi Slum HIV prevalence survey that was carried between September 2006 and November 2007. Ethical clearance for this study was obtained from Ethical and Review committee based in Kenya Medical Research Institute.

### Study site

The study was conducted in Korogocho and Viwandani slums, both located 10 km East of Nairobi City where APHRC runs the Nairobi Urban Health and Demographic surveillance system [NUHDSS]. These two slums are characterized by poor housing, lack of clean water, poor sanitation, unemployment, poverty, and overcrowding. Viwandani slum is located very close to the city’s industrial area and is home to many low income youths working in the industrial area. Korogocho is a more established slum settlement with a high proportion of men living with their spouses and children. Korogocho residents are predominantly either very low-income earners or unemployed. Additionally, residents of Viwandani are relatively more educated than those of Korogocho.

### Study population

Blood samples for anti-HCV, anti-HIV-1 and HBsAg testing were obtained from residents of Korogocho and Viwandani who consented to the study and were registered by the NUHDSS. Participants were aged 15–49 years for females and 15–54 years for males. A structured questionnaire was used to obtain socio-demographic data. Participants were given information about the objectives of the study and informed consent was affirmed by signing a consent form. The interviewer read out the informed consent to those who could not read. Minors [15–17 years old] who agreed to participate signed the informed consent and in addition their guardians also provided consent by appending their signature or thump prints.

### Sample collection and processing

Blood samples drawn by venipuncture were spotted onto S & S Whatmann filter paper [Schleicher-Schuell,Germany], five spots per paper and air dried at room temperature. Dried blood spots were transported by courier service to the laboratory for further testing. For each sample, one blood spot was cut under sterile conditions and dissolved in 500μl of phosphate buffered saline [PBS] in an eppendorf tube. This was vortexed briefly to dissolve the blood specimen into the PBS The eluted sample was used to sequentially test for HIV, HBsAg and HCV. Fifty microlitres of the eluted sample was serologically tested for HIV-1 antibodies using the Determine HIV-1/ HIV-1-2 [Abbott, Japan] and Uni-Gold [Trinity Biotech, NY, USA]. Testing of HBV was done using the KEMRI Hepcell HBsAg (KEMRI, Nairobi, Kenya) rapid test kit according to the manufacturer’s specifications. A confirmation for the presence of HBsAg was done using the DRG ELISA kit [DRG International, Inc, New Jersey, USA)]. Samples that were screened and confirmed positive by KEMRI Hepcell HBsAg rapid kit and DRG ELISA kit for HBsAg were recorded as positive. Testing of anti-HCV antibodies was done using the KemPaC [KEMRI, Nairobi,Kenya] rapid test kit. Confirmation for the presence of anti-HCV antibodies was done using the DRGs’ Hepatitis C Virus diagnostic kit [DRG International, Inc, New Jersey, USA]. Only samples that were screened and confirmed as positive by KemPac test kit and DRGs’ Hepatitis C Virus diagnostic kit were recorded as positive.

### Data management and analysis

All data generated were analyzed using statistical package STATA version 12. Descriptive statistics of socio-demographic variables and other characteristics of the sampled population were computed. Means and SD were calculated for quantitative variables and proportions for categorical variables. OR and 99% CI were calculated for each association. Multiple logistic regression was used for multivariate analysis to determine association between the socio-demographic characteristics and the presence of HBsAg[+], anti-HCV[+] or anti-HIV[+].

## Results

A total of 1308 subjects were successfully sampled for this hepatitis study, of which 418 [32%] were male and 890 [68%] were female. The overall mean age was 29.3 [± 9.3] years ranging from 15 to 54 years. A significant proportion [61.8%] of the study population was married. Most of the participants [71.8%] had either primary school education or no education at all. Table 1 summarizes the socio demographic characteristics of the study population.

**Table 1:** Socio-demographic characteristics of the study population. [n = 1308]

Characteristic	Male (%)	Female (%)	Total
	n = 418	N = 890	n = 1308
**Age group**			
<20	43 (10.2)	163 (18.3)	206 (15.7)
20–24	55 (13.1)	223 (25.1)	278 (21.2)
25–29	78 (18.5)	187 (21.0)	265 (20.2)
30–34	72 (17.2)	117 (13.1)	189 (14.4)
35–39	64 (15.3)	93 (10.4)	157(12)
40–44	44 (10.5)	67 (7.5)	111 (8.4)
45–49	39 (9.3)	40 (4.5)	79 (6)
50+	24 (5.7)	0	24 (1.83)
**Marital status**			
Married	288 (68.9)	522 (58.6)	810 (61.8)
Never married	101 (24.1)	235 (26.4)	336 (25.5)
Divorced/separated/widowed	30 (7.1)	130 (14.6)	163 (12.6)
**Education**			
Primary & below	252 (60.2)	688 (77.3)	940 (71.8)
Secondary	158 (37.7)	198 (22.2)	356 (27.2)
Higher	8 (1.9)	4 (0.4)	12 (0.92)

The prevalence of the three viruses varied between the two informal urban settlements. The overall prevalence of anti-HIV, HBsAg and anti-HCV was 268 (20.4%), 173 (13.3%) and 10 [ [0.76%) respectively. HIV infection was significant higher in participants who were either separated or divorced (45% (OR 1.57 (1.23–2.04) p<0.001). It was however, non-significantly higher in females (21.05%) as compared to males(19.09%). There was also significant HIV infection among age groups 20–24 years old and 30–39 years old (p<0.001)as shown in Table 2. HBV infection was significantly higher among participants from Viwandani (18.58%, p<0.001). They were twice as likely to be HBV infected (OR 2.14 (1.53–3.00) p < 0.001) as compared to those from Korogocho (Table 3). However, there was no statistically significant association between hepatitis B virus infection, marital status, age group and education status. Anti-HCV was higher in females (1%) as compared to males and that females were five times more likely to suffer from HCV as compared to males. Participants from Viwandani were seven times more likely to be HCV infected (1.67%, 7.67 (1.55–37.83) p = 0.012) as compared to those residing in Korogocho. Table 2 shows the prevalence rates of HIV, HBV and HCV.

**Table 2:** Prevalence rates of HIV, HBV and HCV.

Characteristic		HIV			HBV			Anti-HCV		
	Population	% pos	OR(99% CI)	p- value	% pos	OR(99% CI)	p-value	%pos	OR(99% CI)	p-value
Study site	Korogocho	21.50%	1.19 (0.90–1.57)	0.22	10.10%	0.50 (0.36–0.69)	< 0.001	0.24%	0.14 (0.03–0.67)	0.0041
	Viwandani	18.58%	1.19 (0.90–1.57)	0.22	18.58%	0.50 (0.36–0.69)	< 0.001	1.67%	0.14 (0.03–0.67)	0.0041
Gender	Male	19.09%	0.88 (0.66–1.18	0.4	14.08%	1.12 (0.80–1.56)	0.526	0	0.23 (0.03–1.85)	0.134
	Female	21.05%	0.88 (0.66–1.18	0.4	12.88%	1.12 (0.80–1.56)	0.526	1%	0.23 (0.03–1.85)	0.134
Marital status	Divorced	45%	1.57 (1.23–2.04)	<0.001	17.21%	1.32 (0.10–1.57)	0.892	1.65%	0.46 (0.27–2.08)	0.682
	Married	21.97%	1.57 (1.23–2.04)	<0.001	13.01%	1.32 (0.10–1.57)	0.892	0.58%	0.46 (0.27–2.08)	0.682
	Never married	12.87%	1.57 (1.23–2.04)	<0.001	12.58%	1.32 (0.10–1.57)	0.892	0.84%	0.46 (0.27–2.08)	0.682
Education	Dont know	14.29%	0.46 (0.17–0.86)	0.01	28.57%	0.34 (0.12–1.02)	0.816	0.00%	1.17 (0.86–3.12)	0.331
	Never attended	36.84%	0.46 (0.17–0.86)	0.01	17.54%	0.34 (0.12–1.02)	0.816	1.75%	1.17 (0.86–3.12)	0.331
	Higher	0.00%	0.46 (0.17–0.86)	0.01	8.33%	0.34 (0.12–1.02)	0.816	0.00%	1.17 (0.86–3.12)	0.331
	Secondary	16.48%	0.46 (0.17–0.86)	0.01	13.13%	0.34 (0.12–1.02)	0.816	1.12%	1.17 (0.86–3.12)	0.331
	Primary	21.20%	0.46 (0.17–0.86)	0.01	13.02%	0.34 (0.12–1.02)	0.816	0.58%	1.17 (0.86–3.12)	0.331
Age group	20–24 years	14.50%	1.22 (1.03–1.36)	<0.001	11.07%	0.83 (0.66–1.45)	0.87	1.07%	0.89 (0.56–1.42)	0.058
	25–29 years	22%	1.22 (1.03–1.36)	<0.001	13.21%	0.83 (0.66–1.45)	0.87	0.75%	0.89 (0.56–1.42)	0.058
	30–34 years	17.54%	1.22 (1.03–1.36)	<0.001	14.81%	0.83 (0.66–1.45)	0.87	2.65%	0.89 (0.56–1.42)	0.058
	35–39 years	17.10%	1.22 (1.03–1.36)	<0.001	12.66%	0.83 (0.66–1.45)	0.87	0%	0.89 (0.56–1.42)	0.058
	40–44 years	8.96%	1.22 (1.03–1.36)	<0.001	15.32%	0.83 (0.66–1.45)	0.87	0%	0.89 (0.56–1.42)	0.058
	45–49 years	9.30%	1.22 (1.03–1.36)	<0.001	13.92%	0.83 (0.66–1.45)	0.87	0%	0.89 (0.56–1.42)	0.058
	50–54 years	2.99%	1.22 (1.03–1.36)	<0.001	8.33%	0.83 (0.66–1.45)	0.87	0%	0.89 (0.56–1.42)	0.058
	< 20 years	7.46%	1.22 (1.03–1.36)	<0.001	14.56%	0.83 (0.66–1.45)	0.87	0%	0.89 (0.56–1.42)	0.058

**Table 3:** Multivariate analysis between HIV, HBV, HCV and sociodemographic characteristics.

	HIV-1 Infection				HBV Infection				HCV Infection			
	Univariate Analysis		Multivariate Analysis		Univariate Analysis		Multivariate Analysis		Univariate Analysis		Multivariate Analysis	
Variables	Odds Ratio (IQR)	P-value	Odds Ratio (IQR)	P-value	Odds Ratio (IQR)	P-value	Odds Ratio (IQR)	P-value	Odds Ratio (IQR)	P-value	Odds Ratio (IQR)	P-value
Study Site	1.20 (0.90,1.57)	0.218	0.94 (0.69,1.27)	0.675	0.50 (0.36,0.69)	< 0.001	2.14 (1.53,3.00)	< 0.001	0.14 (0.03,0.67)	0.0041	7.67 (1.55,37.83)	0.012
Gender	0.88 (0.66,1.18)	0.396	1.13 (0.82,1.56)	0.472	1.12 (0.80,1.56)	0.526	0.94 (0.65,1.35)	0.727	0.23 (0.03,1.85)	0.134	5.78 (0.69,48.65)	0.108
Marital Status	1.57 (1.23,2.04)	< 0.001	1.98 (1.52,2.57)	< 0.001	1.32 (0.10,1,57)	0.892	1.09 (0.80,1.49)	0.585	0.46 (0.27,2.08)	0.682	1.12 (0.33,3.84)	0.858
Education	0.46 (0.17,0.86)	0.0055	0.72 (0.55,0.93)	0.012	0.34 (0.12,1.02	0.816	0.77 (0.57,1.04)	0.085	1.17 (0.86,3.12)	0.331	1.32 (0.42,4.18)	0.636
Age Group	1.22 (1.03,1.36)	< 0.001	1.14 (1.05,1.23)	0.003	0.83 (0.66,1.45)	0.870	0.99 (0.89,1.10)	0.863	0.89 (0.56,1.42)	0.058	0.97 (0.61,1.52)	0.881

In addition, multivariate logistic regression was performed to determine if there was an association between the infections and demographic characteristics. The study sites and gender did not show any significant association with HIV-1 Infection. However, marital status (divorced or separated) p < 0.001, OR (95% CI) = 1.98 (1.52, 2.57), education (No education) p = 0.012, OR (95% CI) = 0.72 (0.55, 0.93) and age group (20–24, 30–39) p = 0.003, OR (95% CI) = 1.14 (1.05, 1.23) showed significant association with HIV-1 infection. On HBV infection, only study site p<0.001, OR (95% CI) = 2.14 (1.53, 3.00) showed significant association. Gender, marital status, education and age group did not show any significant association with HBV infection. This shows that HBV infection varied between the demographic factors. HCV infection showed a very similar result to HBV infection whereby only study site showed significant association with a p value of 0.012, OR (95% CI) = 7.67 (1.55, 37.83). Table 3 shows the multivariate analysis to determine association of the three infections and the socio-demographic characteristics.

Coinfection of HIV and HBV occurred in 56 individuals (4.26%) while that of HIV-1 and HCV was detected in 6 subjects (0.46%). Only two people out of the 1308 (0.15%) were co-infected with all the three viruses together. Of the HIV-1 positive subjects, 20.9% were positive for HBV. The odds of being infected with HBV were twofold when one is HIV-1 positive (OR 2.08, (1.46–2.97) 95% CI). The likelihood of being infected with HIV-1 when one is a HCV carrier were fivefold than when one is not a carrier (OR 5.93, (1.65–21.30), 99% CI). Four (0.3%) subjects were infected with both HBV and HCV.

## Discussion

Epidemiological studies of HIV, HBV and HCV are crucial in the formulation of preventive strategies and planning of health care programmes. Africa has been hit hardest by the HIV pandemic and has the second highest HBV and HCV prevalence, following Asia [14]. The prevalence rates of these infections vary according to the risk factors involved, socioeconomic status, and initial burden of infectious markers in the community, which vary from one country to another and even between different regions within the same country. Such data are rarely available in African countries [4]. Kenya continues to be vulnerable to threats of HIV/AIDS and other chronic viral infections including HBV and HCV. There is evidence that co-infection with HBV and HCV will contribute significantly to morbidity and mortality within the HIV positive population over the coming years; this may be partly due to increase in survival of HIV-infected patients as a result of accessibility to highly active antiretroviral therapy (HAART) in developing countries [14]. Studies have shown that HBV co-infection in the setting of HIV complicates the clinical course and management of HIV infection.

The HIV-1 prevalence of the study participants from Korogocho and Viwandani was significantly higher than the observed national prevalence. Our results show a high HIV-1 prevalence rate of 20.4% for the sampled population. In Kenya, urban residents have a significantly higher risk of HIV infection (7.2%) than rural residents (6.0%) [15]. Similar findings were reported by a study done by Nyovani *et al* [13] using the same pool of recruited participants which showed the HIV-1 prevalence to be 12% in both slums. However, the sample size used in this particular study was bigger (n = 4767), hence explaining the disparities in the prevalence rates. Other studies by Bigogo *et al* [16] and McKinnon *et al* ([17]) also reported high HIV prevalences of 11% and 40% respectively. The high prevalence rates in these slum areas could be attributed to early initiation of sex, multiple sexual partnerships and low use of condoms.

The overall prevalence of HBV in Korogocho and Viwandani was 13.3%. It was however noted that participants from Viwandani were twice as likely to suffer from HBV infection as compared to those from Korogocho. The high prevalence of HBV in Viwandani could be related to the fact that a majority of Viwandani residents do not stay with their partners, are unemployed and have low income and hence engage in risky sexual behaviour that could make them vulnerable to these infections. A study by Hyams *et al* found that 11.4% of outapatients attending three district hospitals in eastern Kenya were positive for HBsAg [8]. Similarly, a study reported by Zoufaly *et al*, in Cameroon shows a HBsAg positivity rate of 12.6% among patients seeking antiretroviral care [18] and a much higher rate of 44.4% is also reported in a Nigerian study [19]. On the contrary, a lower HBsAg prevalence rate of 6.5% was observed in Bangladesh (1).

We oberved an overall prevalence of HCV of 0.76% which was lower than the 1.5% and 1.8% HCV prevalence rate reported in blood donors from Nairobi [9]. This findings relate to data from a study done on the prevalence of HCV and its genotypes among a cohort of drug users in Kenya which estimates the prevalence in the general population to be between 0.2%-0.9% [20]. Much higher rates of 22.2% and 17.5% are also reported in other studies in Kenya [19], and Egypt [21]. However, one demerit of most anti-HCV studies use blood donors and other high risk groups to report on the frequency of HCV and hence may underestimate the real prevalence of the virus in the general population [22]. In addition,this study had a limitation that should be considered, in that HCVinfection was based on detection by antibodies rather than detection of HCV RNA.

Although co infections with HBV and HCV among HIV positive patients is well documented in developed countries, the demographics and impact of these infections are not well defined in low resource countries like Kenya. Studying patterns of co-infection with HBV, HCV and HIV is of great importance, particularly in the context of controlling morbidity and mortality caused by liver disease. This study observed a lower HBV/HIV dual infection as compared to studies done in Kenya, Nigeria, Ethiopia and South Africa [23, 24,25,10, 26]. We also observed that the odds of being infected with HBV were twofold when one was HIV positive. It is known that HIV coinfection influences the clinical outcomes of patients with HBV infection and therefore accelerates progression of liver disease among them. Although a number of prevalence studies on HIV-1/HBV dual infection have been done within Kenya and in Africa, conflicting results have been observed with both higher and lower rates of HBV being reported in HIV-1- positive patients [27]. HIV-1/HCV dual infection was 0.46% in the general population sampled and 2.2% in HIV-1 positive samples. We also noted that there is high chance of getting HIV-1 when one is HCV positive. These findings are similar with study done in Aga Khan University hospital on HIV-1 positive patients attending the HIV-1 clinic whereby 1% of the patients were found to be co-infected with HIV-1 and HCV [10]. These findings also concur in studies done in Zambia (2.2%), Gambia (0.6%) and Cote d’voire (1.2%) [28,29,30]. However, higher rates of HIV/HCV coinfection are reported in studies done in Nigeria (4.8%) and Malawi (5.7%) [31,32, 33]. Only two samples were found to be co-infected with HIV-1/HBV/HCV. These findings are similar to those reported in Kenya, Ethiopia and Nigeria hence indicating a maintained low rate of these trio infections [23,, 10,, 32s].

We found that the prevalence rate of HBV, HCV and HIV varied with age. Anti-HIV was most prevalent in the age group 30–39 years, followed by the 20–24 years. This could be partly because age is strongly linked to sexual experience, frequency of sex, and risk-taking. Those who were divorced or separated had high chances of being HIV positive. The prevalence of anti-HCV was higher among females. Surprisingly, socio-demographic factors such as age, marital status, and educational attainment were not significantly associated with the risk of being HBV positive.

## Conclusion

HIV and HBV infections are common in urban slum establishments and might become a major health problem in future if control measures are not put in place. Periodic checkups and health education are required for better control strategies.
