# Correction: Hepatitis B, Hepatitis C and HIV-1 Coinfection in Two Informal Urban Settlements in Nairobi, Kenya

**DOI:** 10.1371/journal.pone.0133342

**Published:** 2015-07-20

**Authors:** Glennah Kerubo, Samoel Khamadi, Vincent Okoth, Nyovani Madise, Alex Ezeh, Abdhalah Ziraba, Matilu Mwau

The sixth author’s name is spelled incorrectly. The correct name is: Abdhalah Ziraba. The correct citation is: Kerubo G, Khamadi S, Okoth V, Madise N, Ezeh A, Ziraba A, et al. (2015) Hepatitis B, Hepatitis C and HIV-1 Coinfection in Two Informal Urban Settlements in Nairobi, Kenya. PLoS ONE 10(6): e0129247. doi:10.1371/journal.pone.0129247.
